# Increased unbound drug fraction in acute carbamazepine intoxication: suitability and effectiveness of high-flux haemodialysis

**DOI:** 10.1007/s00134-012-2501-8

**Published:** 2012-02-11

**Authors:** M. A. Sikma, M. P. H. van den Broek, J. Meulenbelt

**Affiliations:** 1 Department of Intensive Care, University Medical Centre Utrecht, P.O. Box 85500, 3508 GA Utrecht, The Netherlands; 2 Department of Clinical Pharmacy, University Medical Centre Utrecht, P.O. Box 85500, 3508 GA Utrecht, The Netherlands

Dear Editor,

A 61-year-old woman (patient A) was admitted to our intensive care unit with carbamazepine overdose. Carbamazepine total plasma concentration determined by enzyme-multiplied immunoassay technique (EMIT) was 52.5 mg/L (reference 4–12 mg/L) of which 22.7 mg/L was unbound (43%). Unbound carbamazepine was determined in plasma by EMIT after ultrafiltration (Amicon Ultra centrifugal filter (Millipore), 2,500 rpm, 30 min at 25°C). Despite supportive care (pacemaker, inotropes and vasopressors) and multiple activated charcoal administration, the patient died shortly after admission as a result of refractory shock.

**Fig. 1:**
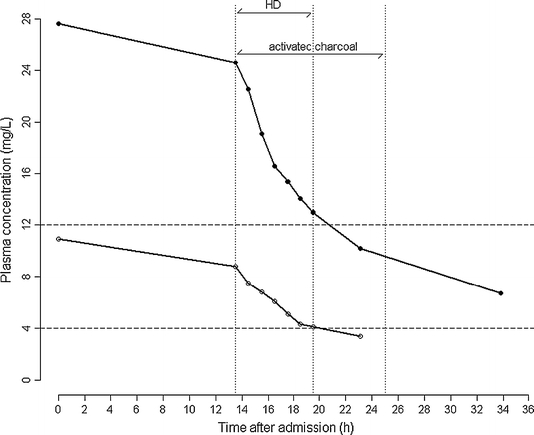
Carbamazepine total (*solid circles*) and unbound (*open circles*) plasma concentrations in patient B. Therapeutic range for total plasma concentrations 4–12 mg/L

Soon after, a 41-year-old man (patient B) was admitted after resuscitation due to ventricular fibrillation. For epilepsy he was treated with carbamazepine (400 mg 5 times/day). As a result of repeated ventricular fibrillation, intravenous amiodarone (300 mg/day) was started. Two days later a deep coma developed. Because of a high daily dose of carbamazepine and its interaction with amiodarone, we suspected a carbamazepine intoxication. Total carbamazepine plasma concentration was 27.4 mg/L of which 10.9 mg/L was unbound (40%). Carbamazepine intoxication was treated with multiple-dose activated charcoal and high-flux haemodialysis (HF-HD); filter Fresenius Helixon^®^ FX1000, blood flow 350 mL/min, dialysate flow 500 mL/min, ultrafiltration rate 120 mL/h, ultrafiltration coefficient 75 mL/(h mmHg). Carbamazepine plasma concentration decreased to 13.0 mg/L (4.1 mg/L unbound; 32%) (Fig. 1). The patient fully recovered.

Treatment of carbamazepine intoxication consists of supportive care, prevention of further absorption and enhancement of elimination via haemoperfusion [1, 2]. But haemoperfusion has serious adverse effects and the facilities are often not available [1, 3]. Effectiveness of haemodialysis depends on drug characteristics, dialysis system properties and dialysis conditions. Haemodialysis in carbamazepine overdose is considered not to be efficacious because of low hydrophilicity (Log* P* = 1.98) and the high degree of protein binding (70–80%), although carbamazepine is small enough for filtration (236 Da) and has a low distribution volume (0.8–1.8 L/kg) [1].

Elimination rate constants (*k*), calculated as *k* = ln 2/half-life (h), reflect the effectiveness of the elimination route. Overall *k* is defined as the sum of all individual *k* values. Calculated individual *k* values in patient B were 0.009/h for endogenous metabolism, 0.059/h for dialysis (including membrane adsorption) and 0.039/h for charcoal treatment. Endogenous *k* was calculated from the time period in which no activated charcoal or HF-HD was applied. *k* of activated charcoal was calculated by subtracting *k* endogenous from *k* overall when only endogenous clearance and activated charcoal therapy were present. *k* dialysis is *k* overall during HF-HD minus *k* endogenous and *k* activated charcoal. Limited blood sampling during the initial rapid clearance of free drug from the plasma by HF-HD may partly hamper the interpretation of the results. However, on the basis of the half-life initially and later on during HF-HD, we assume that no significant rapid initial phase took place (see Fig. 1).

HF-HD was more effective than multiple activated charcoal treatment alone, whereas the combination of both was about ten times more effective than endogenous clearance. Probably, endogenous *k* was decreased because of the presence of amiodarone, a CYP3A4 inhibitor [4]. Owing to the increased unbound drug concentration in carbamazepine intoxication, HF-HD proved to be an effective extracorporeal elimination technique.

In conclusion, HF-HD is an effective extracorporeal elimination technique in carbamazepine intoxication as a result of the increased unbound drug fraction and can replace haemoperfusion.
